# The effects of probiotics, prebiotics, and synbiotics on aversive memory in a premature ovarian failure model in rat

**DOI:** 10.14814/phy2.70480

**Published:** 2025-08-19

**Authors:** Mohammad Hossein Madahali, Rasul Saberi, Sareh Karimi, Arezoo Rajabian, Mahmoud Hosseini

**Affiliations:** ^1^ Department of Anatomy and Cell Biology School of Medicine, Mashhad University of Medical Sciences Mashhad Iran; ^2^ Department of Anatomical Sciences and Reproductive Biology School of Medicine, Isfahan University of Medical Sciences Isfahan Iran; ^3^ Applied Biomedical Research Center Mashhad University of Medical Sciences Mashhad Iran; ^4^ Psychiatry and Behavioral Sciences Research Center Mashhad University of Medical Sciences Mashhad Iran; ^5^ Sterility and Reproductive Biology Research Center Emam Reza Hospital, Mashhad University of Medical Sciences Mashhad Iran; ^6^ Neuroscience Research Center Mashhad University of Medical Sciences Mashhad Iran; ^7^ Department of Neuroscience, Faculty of Medicine Mashhad University of Medical Sciences Mashhad Iran; ^8^ Department of Physiology School of Medicine, Mashhad University of Medical Sciences Mashhad Iran

**Keywords:** acetylcholinesterase, cognition, oxidative stress, prebiotics, premature ovarian failure, probiotics, synbiotics

## Abstract

Cognitive impairment occurs in cisplatin‐induced premature ovarian failure (POF). The impacts of probiotics, prebiotics, and synbiotics on passive avoidance memory, oxidative stress markers, and acetylcholinesterase (AChE) activity in the POF model were examined. POF was induced by cisplatin (2 mg/kg, IP/7 days). In the POF + Probiotic, POF + Prebiotic, and POF + Synbiotic groups, the treatments were done during 28 days after 7 days of cisplatin injection. The POF model was confirmed by a decrease in developing follicles. A decrease in latency in the passive avoidance test was seen in the POF group (*p* < 0.05–*p* < 0.001) along with a decrease in thiol and superoxide dismutase (SOD) and an increase in malondialdehyde (MDA) and AChE (*p* < 0.01–*p* < 0.001). An improvement in latency time in the passive avoidance test was seen in all POF + Probiotic, POF + Prebiotic, and POF + Synbiotic groups (*p* < 0.05–*p* < 0.001). Treatment with probiotics, prebiotics, and synbiotics improved thiol, SOD, and attenuated AChE activity in the hippocampus, cortex, and cerebellum (*p* < 0.05–*p* < 0.001). Our results confirm that probiotics, prebiotics, and their combination as synbiotics have protective effects against oxidative stress‐related brain injury and improve aversive memory and cholinergic function in a POF model in rats.

## 1 INTRODUCTION

It is widely recognized that women are nearly twice as likely as men to develop Alzheimer's disease (AD). This increased susceptibility may be attributed to the fact that women generally have a longer lifespan than men. Additionally, hormonal changes associated with the reproductive system could contribute to this elevated risk (Tarrá Marrugo, 2025). Premature ovarian failure (POF) is marked by increased levels of gonadotropin hormones and decreased levels of estrogen in females younger than 40 years old, and it is emerging as a significant public health concern (Jankowska, 2017; Panay et al., 2020). The estimated prevalence rate of POF is approximately 3.7% (Golezar et al., 2019). POF is associated with neurodevelopmental challenges and an increased risk of dementia. Furthermore, evidence suggests that surgical menopause induced by bilateral oophorectomy before the age of 45 is linked to a higher risk of dementia and cognitive decline, particularly in memory‐related functions (Soni & Hogervorst, 2014). It is shown that early ovarian failure, regardless of the age at which it occurs, is associated with a faster decline in verbal and semantic memory, as well as processing speed (Georgakis et al., 2019). Another study found that POF is linked to long‐term detrimental effects on cognitive abilities, which are only partially mitigated by hormone therapy (Ryan et al., 2014). POF, also referred to as premature menopause, impacts the central nervous system (CNS) in multiple ways, leading to cognitive impairments and potentially increasing the risk of dementia over time. These effects are primarily attributed to decreased estrogen levels and may be further influenced by cardiovascular risks, autoimmune conditions, and the natural aging process. Women with POF or early menopause are also at a higher risk of developing other health conditions (Sochocka et al., 2023). Additionally, findings from a study suggest that certain biomarkers of inflammation and AD are altered in women with POF (Liu et al., 2020; Marongiu, 2019).

Cisplatin is a potent chemotherapeutic agent used to treat various types of cancers, including those of the breast, ovaries, lungs, bladder, head and neck, testicles, esophagus, cervix, brain, and lymph nodes (Ho et al., 2016). Although cisplatin therapy is effective in destroying cancer cells, it also adversely affects healthy tissues (Barabas et al., 2008). Studies have shown that cisplatin can cause detrimental effects on the ovaries, particularly damaging primordial follicles. This ovarian damage may result in a reduced ovarian reserve, POF, and early onset of menopause (Morgan et al., 2013).

Research on host nutrition and metabolism has significantly expanded over the past few decades (Ejtahed et al., 2016). Prebiotics are dietary and nondigestible carbohydrates that can promote the growth and function of beneficial microorganisms in the gut (Gibson et al., 2017). Synbiotics are a combination of probiotics and prebiotics. They synergistically work to support the maintenance of beneficial bacteria in the gut (Pandey et al., 2015). These compounds are believed to positively influence the metabolism of the host (Holscher, 2017). Additionally, probiotics produce bacteriocins and their metabolites, including acetic and lactic acids, and they suppress pathogen development employing antimicrobial effects and pH changes (Gillor et al., 2008). Probiotics like *Bifidobacterium infantis* and *Bifidobacterium brucella* can stimulate intestinal dendritic cells by interacting with toll‐like receptors and initiating retinoic acid metabolism (Konieczna et al., 2012). This stimulation leads to the formation of various T cells and the secretion of interleukin (IL)‐10. Conversely, specific probiotic strains may trigger an immune response that promotes inflammation, enhancing the function of natural killer cells and their ability to eliminate harmful pathogens through phagocytosis (Rocha‐Ramírez et al., 2017). Synbiotics exhibit immunomodulatory and anti‐inflammatory effects, as well as antimutagenic, antigenotoxic, and anticarcinogenic properties (Lo et al., 2004). These compounds are also known to provide protective benefits against genitourinary tract infections (Zuccotti et al., 2008). Probiotics can significantly impact mucosal immunity by influencing different host cells that are involved in immune responses (Zhang et al., 2007). Moreover, probiotics have been shown to have the capacity to be a protection against intestinal damages and ovarian dysfunction induced by cisplatin and display anti‐inflammatory and anti‐oxidant characteristics (Tang et al., 2024; Madahali et al., 2025). Most commercial probiotic products are considered to be safe, and they may enhance host well‐being. Furthermore, certain probiotic strains could serve as a complementary approach in the prevention and/or management of cancer by modulating intestinal microbiota and immune responses (Wu et al., 2021).

Findings from research studies suggest that incorporating probiotics and prebiotics into the diet is associated with enhanced cognitive skills in elderly individuals, particularly those with specific health conditions (Freijy et al., 2024; Heyck & Ibarra, 2019; Kim et al., 2021). Results from an unbiased study demonstrated that the use of probiotics and prebiotics significantly improved cognitive skills and memory. These enhancements were achieved by increasing brain‐derived neurotrophic factor (BDNF) levels and reducing neuroinflammatory responses in the hippocampus of mice with cognitive impairment (Ton et al., 2020). Another study found that interventions targeting gut microbiota improvement through probiotic supplementation can significantly enhance cognitive abilities and daily living skills in individuals with cognitive deficits and AD (Heyck & Ibarra, 2019). Additionally, the findings of another study indicated that incorporating synbiotic supplementation could potentially alleviate age‐related memory decline (Fekete et al., 2024). Neuroprotective and learning and memory improvement of synbiotics in a rat model of stroke was attributed to its anti‐inflammatory effect and its ability to improve BDNF (Cruz‐Martínez et al., 2024). A different study revealed a notable connection between gut microbiota and brain function, discovering that the administration of synbiotics resulted in a significant enhancement in neuronal plasticity, which in turn improved memory and learning in rats (Romo‐Araiza et al., 2023). The current research will examine the impacts of probiotics, prebiotics, and synbiotics on aversive memory in the POF model in rats.

## 2 MATERIALS AND METHODS

### 2.1 Animals and ethical statement

In this research, 38 female rats, around 8–10 weeks in age and weighing between 200 and 250 g, were acquired from the Laboratory Animal Care Center at the Faculty of Medicine, Mashhad University of Medical Sciences. They were kept under standard conditions including regulated temperature at 22–24°C, humidity 55%–65%, and periodic light; the lights were ON at 6 am. The animals were able to freely receive food and water. The standard foods for the rodents were obtained from Javaneh Khorasan Company, Mashhad, Iran. Before the commencement of the study, the rats were co‐housed for 2 weeks to synchronize the estrous cycle in rats (McClintock, 1978; Stiles et al., 2013). The research methods were granted official approval by the Ethics Committee for Animal Experimentation, following the endorsed ethical code IR. MUMS. REC. 1403. 140.

### 2.2 Chemicals

The probiotic strain tested in the trials originated from Yomogi capsules (LOT NO: 2112), approved by Health Canada, and provided by Irenic Pharmaceutical Company Iran. The prebiotic component was sourced from Inulin (LOT NO: 2021014874), which is a fibrelle company and is located in Istanbul, Turkey. A synbiotic blend was formulated by combining probiotics and prebiotics. Furthermore, a model of POF was developed using the chemotherapy drug cisplatin (GTIN:06260607810431, IRC: 7543958057252561) from Sobhan Darou Company, Iran.

### 2.3 Experimental design

The animals were divided into five groups of 6–8 each in a random way.

1. Control group (*n* = 8): To control for potential confounding effects of injection‐related stress, in the control rats, normal saline was injected as the solvent for cisplatin for the same number of days as the drug injection. Additionally, water was given via gavage for 28 days.

2. POF group (*n* = 6): This group with ovarian failure was administered cisplatin (Du et al., 2023), which was diluted to a dosage of 2 mg/kg b.w., and injected intraperitoneally for 7 days. After this, water was given via gavage for 28 days.

3. Probiotic treatment group (POF + Probiotic) (*n* = 8): Following the establishment of the model, the animals received daily gavage of 1 × 10^9^ CFU of Saccharomyces boulardii bacteria (Yumogi capsule) for 28 days (Sevim et al., 2023).

4. Prebiotic treatment group (POF + Prebiotic) (*n* = 8): After establishing the model, animals were given daily gavage of 500 mg/kg body weight of inulin (prebiotic sachet) for 28 days (Xue et al., 2019).

5. Synbiotic treatment group (POF + Synbiotic) (*n* = 8): After model establishment, animals received daily gavage of 1 × 10^9^ CFU of Saccharomyces boulardii bacteria along with 500 mg/kg b.w. of inulin for 28 days.

### 2.4 Tissue collection

Following the completion of the behavioral tasks, the animals were anesthetized using high doses of ketamine–xylazine (100 and 10 mg/ kg respectively). The rats were then sacrificed using carbon dioxide (CO_2_) exposure. The brains were then rapidly dissected on an ice surface, and the cortex, cerebellum, and hippocampus were isolated for analysis. The isolated tissues were blended in ice‐cold PBS and centrifuged at 3000 rpm for 15 min to separate the supernatants. The resultant supernatants were collected for biochemical evaluations (Akbarian et al., 2023; Mokhtari‐Zaer et al., 2020). The ovaries were also collected and suspended in the formalin solution.

### 2.5 Histological assessments

To evaluate and confirm the model of cisplatin‐induced POF, the collected ovarian tissues were stored in 10% formalin for 72 h after the animals were sacrificed. After fixation, the tissues underwent dehydration in alcohol, were cleared in xylene, and subsequently embedded in paraffin. After the blocks were prepared, the ovarian tissues were cut into consecutive sections that were 5 μm thick with the use of a microtome. Ultimately, the slides that were created were stained with hematoxylin and eosin and examined under a light microscope (OLYMPOS BX51).

### 2.6 Biochemical assessments

#### 2.6.1 Measurement of malondialdehyde (MDA) level

As a key marker of lipid peroxidation, the level of MDA was assessed in the tissues. The thiobarbituric acid reactive substances assay was used to measure the amounts of MDA. In summary, tissue homogenates were combined with a solution made up of trichloroacetic acid (Merk Company, Catalog NO: 27242), hydrochloric acid (HCl, Scharlab S.L., Catalog NO: 2782BATCH 13840510), and thiobarbituric acid (TBA, Merck Company, Catalog NO: 1.08180.0025). After incubating the samples in boiling water for 40 min, the samples were centrifuged at 3000 rpm for 5 min. Finally, the absorbance of the supernatants was measured at the wavelength of 535 nm by using a UV–vis spectrophotometer. The MDA concentration was determined according to the previous research (Akbarian et al., 2023).

#### 2.6.2 Evaluation of total thiol groups

For measuring thiol content (or sulfhydryl (SH) groups), Ellman's method was utilized. The DTNB (5,5′‐dithiobis‐(2‐nitrobenzoic acid) Merck Company, Catalog NO: 1.03291.0001) reagent was utilized to measure the total amount of thiol groups. This chemical reaction with sulfhydryl (SH) groups creates a yellow complex that exhibits a peak absorbance at 412 nm. To assess the thiol content, 1 mL of tris (Merck Company, Catalog NO: 1.08387.0500)‐EDTA (ethylenediaminetetraacetic acid, Merck Company, Catalog NO: 1.08387.0500) buffer (30 mM Tris, 3 mM EDTA, pH 8. 2) was combined with 50 μL of the sample, and the starting absorbance (A1) was measured at 412 nm using a UV–vis spectrophotometer. Notably, Tris‐EDTA buffer serves as the blank. Subsequently, 20 μL of the DTNB reagent (dissolved in methanol) was mixed with each sample, and the second absorbance was recorded (A2). The total amount of thiol was expressed as μM per each gram of tissue (Hosseini et al., 2022; Rastegar‐Moghaddam et al., 2022).

#### 2.6.3 Assessment of superoxide dismutase (SOD) activity

SOD activity was measured using the colorimetric method described by Madesh and Balasubramanian (1998). In summary, cortex, hippocampus, and cerebellum homogenates were subjected to pyrogallol (pyrogallol (Tokyo Chemical Industry Company, Japan, Catalog NO: P0570)) and MTT (3‐(4, 5‐dimethylthiazol‐2‐yl) 2, 5‐diphenyltetrazolium bromide) (Tinab shimi, Catalog No. T29809301) solutions during incubation. The reaction was terminated by the addition of dimethyl sulfoxide (DMSO). The mixture was subsequently incubated for 5 min at room temperature, and the absorbance was recorded at 570 nm. One unit of SOD activity was characterized as the amount of enzyme that produces a 50% decrease in the MTT reduction rate (Eshaghi Ghalibaf et al., 2023).

#### 2.6.4 Acetylcholinesterase (AChE) assessment

AChE activity was determined using Ellman's method in the cortical, hippocampal, and cerebellar tissue hemogenates (Hosseini et al., 2024). The evaluation of AChE enzyme activity was carried out using a solution of acetylthiocholine iodide (Sigma Aldrich Company, USA, Catalog No. A5751‐1G). For this aim, each sample was transferred into a cuvette containing DTNB and PBS solution. Following that, the mixture was incubated for 15 min. The reaction is started by the addition of the acetylthiocholine iodide solution. Finally, the absorbance was measured using a UV–vis spectrophotometer at a wavelength of 412 nm. The activities of the enzymes are represented in terms of nanomoles of substrate per each gram of tissue weight (Akbarian et al., 2023; Bardaghi et al., 2023).

#### 2.6.5 Passive avoidance (PA) test

The aversive memory capabilities were evaluated utilizing the PA test. It included a device that featured two equally sized sections, one illuminated and one shaded. In this device, a guillotine door acted as the divider between the two parts. The evaluation of PA capability includes three stages. In the initial phase of the experiment, which lasted for two successive days, the rat was situated in the apparatus to acclimate to it. In the second phase, referred to as the acquisition trial, the rats were once more positioned in the apparatus. The door fell shut on its own immediately after the rat made its way into the dim compartment, at which moment an electric foot shock (2 mA for 2 s) was administered. Finally, the third phase, referred to as the retention trials, took place 3, 24, 48, and 72 h following the training trial, utilizing the same training program. The delay value was determined by timing how long it took for the animal to access the dark compartment. Additionally, the duration of time spent in both the illuminated and shaded areas (in seconds) during 300 s was noted at 3, 24, 48, and 72 h following the administration of the punishment (Akbarian et al., 2023; Mokhtari‐Zaer et al., 2020).

### 2.7 Statistical analysis

All data were examined utilizing SPSS software (version 26) through one‐way ANOVA accompanied by Tukey's post hoc comparisons test and presented as mean ± SD.

The data of passive avoidance test were analyzed using the Kruskal–Wallis followed by Dunn's pairwise comparison test and presented as median and interquartile range. *p* < 0.05 was considered statistically significant. The graphs were drawn using GraphPad Prism software (version 9).

## 3 RESULTS

### 3.1 Results of histological assessments

**FIGURE 1:**
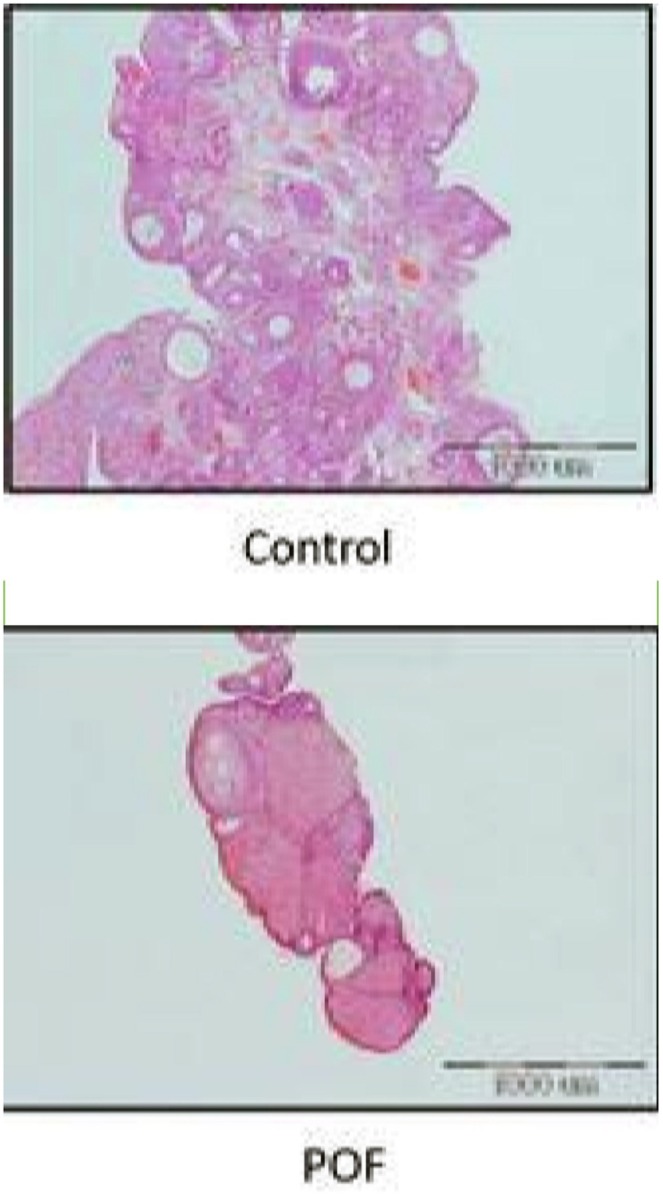
The results of histological examination of ovarian tissue (Scale Bar: 1000 μm).

To confirm the POF model in animals, histology by hematoxylin–eosin staining was employed to examine the primordial and growing follicles. After preparing the tissue slides from each group, primordial, primary, secondary, and atretic follicles were analyzed. The results of our study, as displayed in Figure 1, indicated that the quantity of developing follicles in the POF group was decreased in comparison to the control group. Overall, the process of folliculogenesis was diminished.

### 3.2 Biochemical parameters

#### 3.2.1 Results of examining the levels of oxidative stress factors and AChE activity in the hippocampus

**FIGURE 2:**
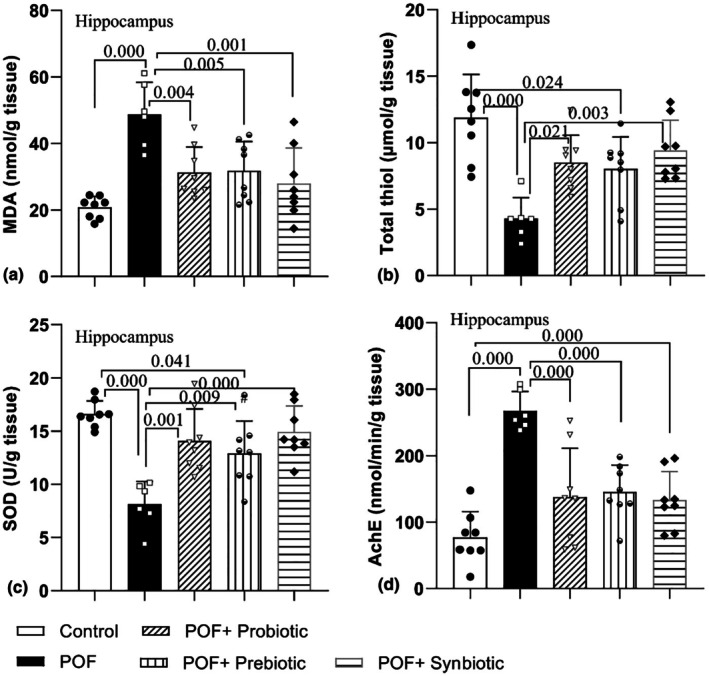
Comparison of the MDA (a) and thiol concentration (b) and SOD (c) and AChE (d) activity in hippocampus tissues between experimental groups. Data are presented as mean ± SD (*n* = 6–8 in each group).

MDA concentration in the hippocampus had a considerable difference among the groups (*F*_4,33_ = 10.35, *p* = 0.000). The results also exhibited that POF increased the level of this product in the hippocampal tissue of rats compared to the control group (*p* = 0.000). With respect to the POF group, the level of MDA in the hippocampal tissue in POF + Probiotic, POF + Prebiotic, and POF + Synbiotic groups significantly declined (*p* = 0.004, *p* = 0.005, and *p* = 0.001, respectively) (Figure 2a).

The thiol level in the hippocampus had a considerable difference among the groups (*F*_4,33_ = 8.87, *p* = 0.000). As illustrated in Figure 2b, POF decreased the total thiol level in the hippocampus of the POF group compared to the control group (*p* = 0.000). Additionally, a notably lower level was noted in the POF + Prebiotic group regarding the total thiol content when compared to the control group (*p* = 0.024) and there was no significant difference between the POF + Prebiotic group and the POF group (*p* = 0.050). This antioxidant parameter significantly was increased in the hippocampus tissue of rats in POF + Probiotic and POF + Synbiotic versus the POF group (*p* = 0.021 and *p* = 0.003, respectively).

SOD activity also varied significantly among the groups in the hippocampus (*F*_4,33_ = 11.06, *p* = 0.000). The findings elucidated that POF was able to lower the activity of this enzyme in the hippocampal tissues in the POF group when it was compared to the control group (*p* = 0.000). Furthermore, a notably lower level was noted in the POF + Prebiotic group regarding SOD activity when it was compared to the control group (*p* = 0.041). This antioxidant parameter was significantly increased in the hippocampus tissue of rats in POF + Prebiotic, POF + Probiotic, and POF + Synbiotic groups versus the POF group (*p* = 0.001, *p* = 0.009, and *p* = 0.000 respectively) (Figure 2c).

The findings from the examination of AChE activity in the hippocampus of rats indicated a significant difference (*F*_4,33_ = 13.86, *p* = 0.000). It was also revealed that AChE activity in the POF group was considerably greater than that observed in the control group (*p* = 0.000). Treatment with probiotics, prebiotics, and synbiotics decreased AChE activity in the treatment groups when compared to the POF group (*p* = 0.000) (Figure 2d).

#### 3.2.2 Results of examining the levels of oxidative stress factors and AChE activity in the cortex

**FIGURE 3:**
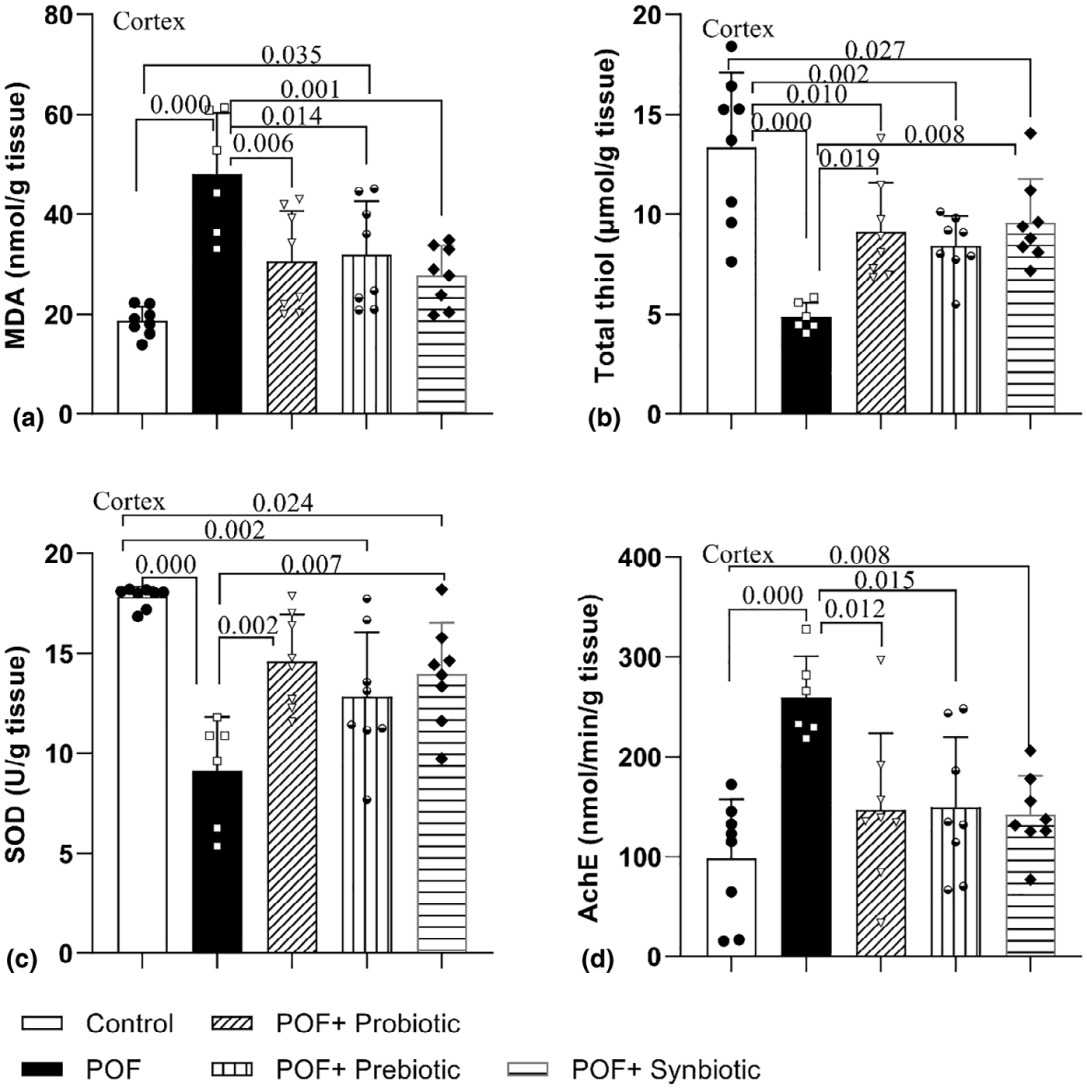
Comparison of the MDA (a) and thiol concentration (b) and SOD (c) and AChE (d) activity in Cortex tissues between experimental groups. Data are presented as mean ± SD (*n* = 6–8 in each group).

The level of MDA in the cortex showed considerable differences among the groups (*F*_4,33_ = 9.93, *p* = 0.000). The findings also showed that POF induction raised the levels of this compound in the cortex tissue of rats in comparison to the control group (*p* = 0.000). The MDA level in the cortical tissue of the POF + Prebiotic group was also greater than that of the control group (*p* = 0.035). Additionally, the MDA levels in the cortex tissue of the POF + Probiotic, POF + Prebiotic, and POF + Synbiotic groups were markedly reduced compared to the POF group (*p* = 0.006, *p* < 0.014, and *p* = 0.001, respectively) (Figure 3a).

The levels of thiol in the cortex also exhibited notable variations between the groups (*F*_4,33_ = 11.18, *p* = 0.000). As shown in Figure 3b, POF decreased the total thiol levels in the cortex of the POF group in comparison to the control group (*p* = 0.000). Furthermore, this antioxidant measure was considerably lower in the cortex tissue of the rats in the POF + Probiotic, POF + Prebiotic, and POF + Synbiotic groups when compared to the control group (*p* = 0.010, *p* = 0.002, and *p* = 0.027, respectively). In addition, the total thiol concentration in the POF + Probiotic and POF + Synbiotic groups showed a notable rise in comparison to the POF group (*p* = 0.019 and *p* = 0.008, respectively) but no significant difference was observed between POF + Prebiotic and the POF group (*p* = 0.068) (Figure 3b).

SOD activity also showed considerable variations among the groups in the cortex (*F*_4,33_ = 11.57, *p* = 0.000). The results showed that POF diminished the enzyme activity in the cortex tissues of the POF group in comparison to the control group (*p* = 0.000). Additionally, the SOD activity in the POF + Prebiotic and POF + Synbiotic groups demonstrated a notably reduced level in comparison to the control group (*p* = 0.002 and *p* < 0.024, respectively). Furthermore, the activity of SOD in the POF + Probiotic and POF + Synbiotic groups was markedly elevated in comparison to the POF model group (*p* = 0.002 and *p* = 0.007, respectively) (Figure 3c).

The examination of AChE activity in the cortex of rats indicated a significant difference (*F*_4,33_ = 6.41, *p* = 0.001). In addition, AChE activity in the POF group was notably elevated compared to that in the control group (*p* = 0.000). Treatment with probiotics, prebiotics, and synbiotics decreased AChE activity in the treatment groups when compared to the POF group (*p* = 0.012, *p* = 0.015, and *p* = 0.008, respectively) (Figure 3d).

#### 3.2.3 Results of examining the levels of oxidative stress factors and AChE activity in the cerebellum

**FIGURE 4:**
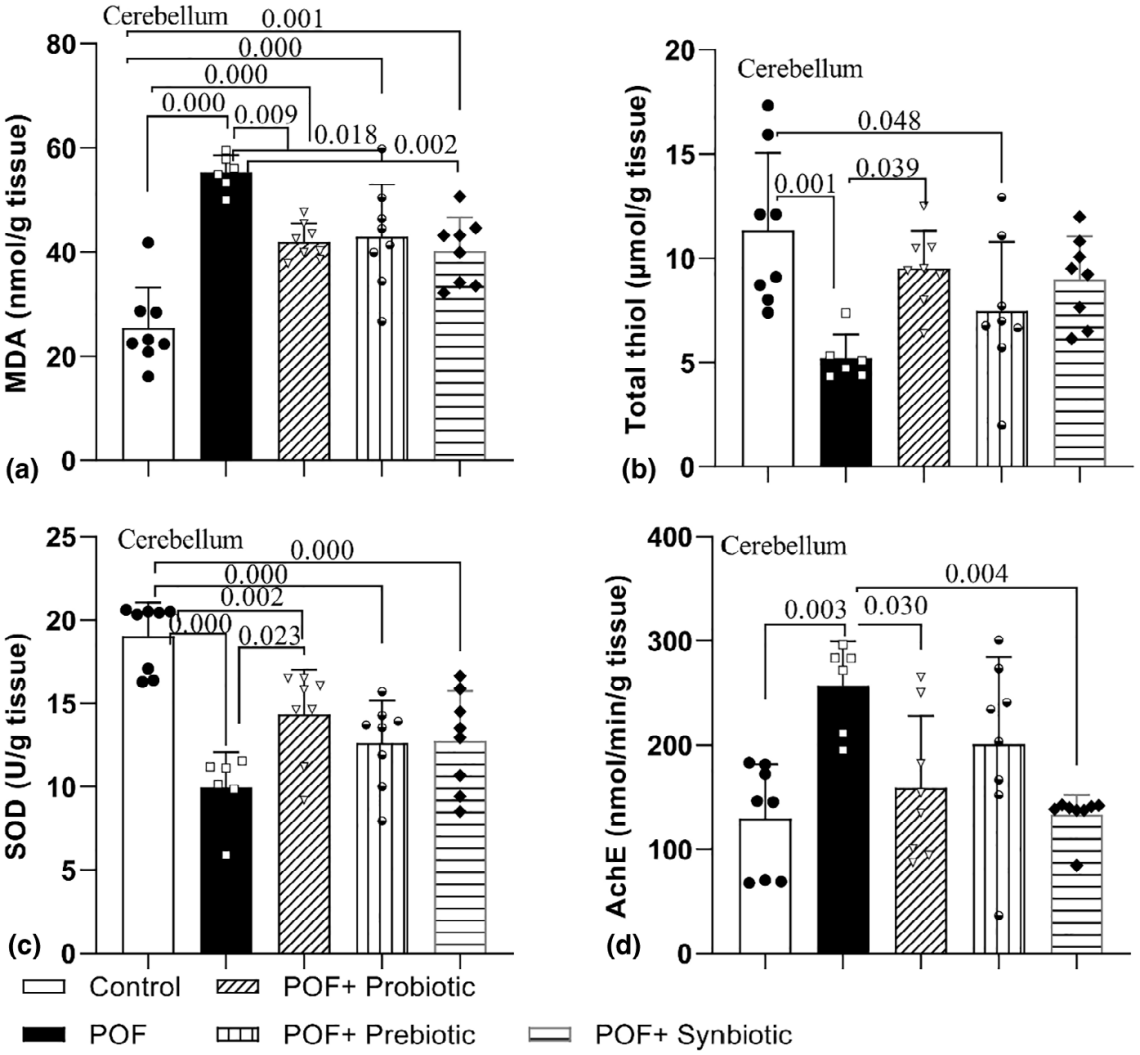
Comparison of the MDA (a) and thiol concentration (b) and SOD (c) and AChE (d) activity in Cortex tissues between experimental groups. Data are presented as mean ± SD (*n* = 6–8 in each group).

The concentration of MDA in the cerebellum differed significantly among the groups (*F*_4,33_ = 16.80, *p* = 0.000). The findings showed that POF induction raised the concentration of MDA in the cerebellum tissue of rats in comparison to the control group (*p* = 0.000). In all treatment groups, a significantly high level of MDA concentration was seen when it was compared to the control group (*p* = 0.000, *p* = 0.000, and *p* = 0.001). The amounts of MDA within the cerebellum tissue were considerably lowered in the POF + Probiotic, POF + Prebiotic, and POF + Synbiotic groups compared to the POF model group (*p* = 0.009, *p* = 0.018, and *p* = 0.002, respectively) (Figure 4a).

Moreover, the levels of thiols in the cerebellum showed notable discrepancies among the groups (*F*_4,33_ = 5.18, *p* = 0.002). As illustrated in Figure 4b, POF decreased the thiol levels in the POF group when compared to the control group (*p* = 0.001). A notable low level of thiol content was also noticed in the POF + Prebiotic group in comparison to the control group (*p* = 0.048). In contrast, this antioxidant factor demonstrated a notable rise in the cerebellum tissue of rats within the POF + Probiotic group when compared to the POF group (*p* = 0.039). There was no significant difference between the POF + Prebiotic and POF group (*p* = 0.521) and also between the POF + Synbiotic and POF group (*p* = 0.088).

SOD activity also varied significantly among the groups in the cerebellum (*F*_4,33_ = 12.90, *p* = 0.000). The results indicated that POF diminished the function of this enzyme in the cerebellum tissues of the POF, POF + Probiotic, POF + Prebiotic, and POF + Synbiotic groups in comparison to the control group (*p* = 0.000, *p* = 0.006, *p* = 0.000, and *p* = 0.000 respectively). This antioxidant indicator exhibited a considerable rise in the cerebellum tissue of rats in the POF + Probiotic group when contrasted with the POF group (*p* = 0.023) (Figure 4c). There was no significant difference between the POF + Prebiotic and POF groups and also between the POF + Synbiotic and POF groups (Figure 4c).

The examination of acetylcholinesterase (AChE) activity in the cerebellum of rats of the groups revealed a significant difference (*F*_4,33_ = 5.70, *p* = 0.001). It also showed that AChE activity in the POF group was markedly greater than that in the control group (*p* = 0.003). Treatment using probiotics and synbiotics decreased AChE activity in the treatment groups in comparison to the POF group (*p* = 0.030 and *p* = 0.004, respectively) but there was no significant difference between the POF + Synbiotic and POF group (Figure 4d).

### 3.3 PA test results

**FIGURE 5:**
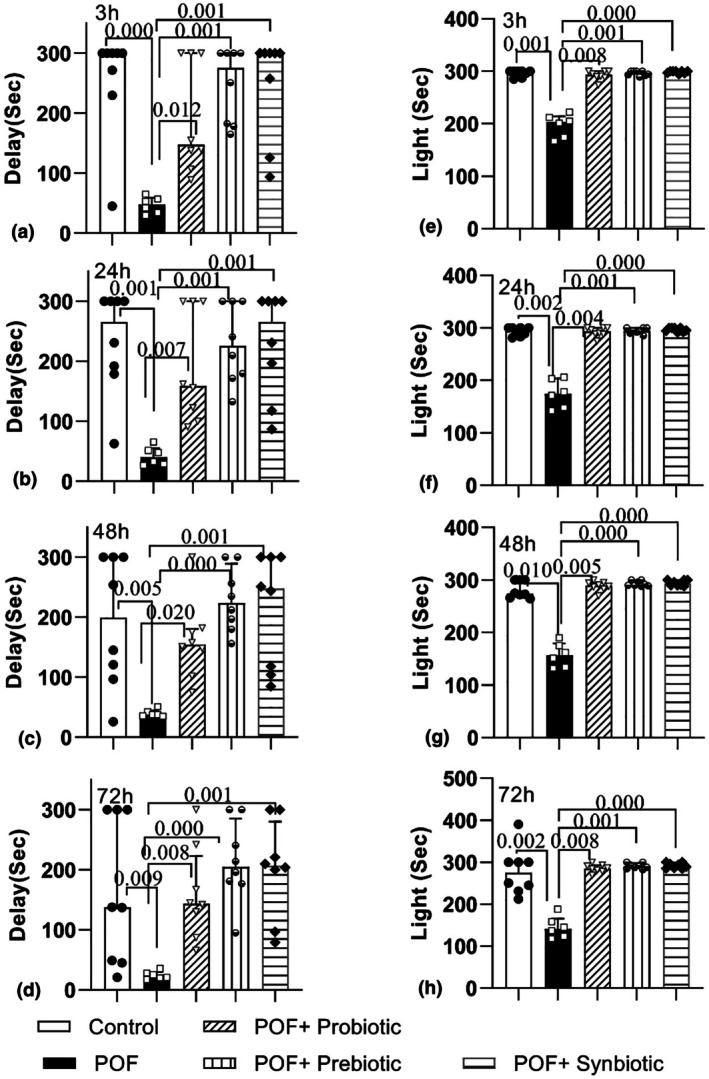
Comparison of the time delay to enter the dark room (a, b, c, d) and the total time spent in the lightroom (e, f, g, h), at the 3, 24, 48, and 72 h after receiving a shock in the passive avoidance test in the experimental groups. The data are presented as median and interquartile range (*n* = 6–8).

As illustrated in Figure 5a–d, the latency in entering the dark region in the POF group was significantly reduced in comparison to the control group at 3, 24, 48, and 72 h following shock delivery (*p* = 0.000, *p* = 0.001, *p* = 0.005, and *p* = 0.009 for 3, 24, 48, and 72 h, respectively). With the POF group, administration of probiotics, prebiotics, and synbiotics notably enhanced the delay time in the treatment groups at 3 h (*p* = 0.012, *p* = 0.001, and *p* = 0.001, respectively), 24 h (*p* = 0.007, *p* = 0.001, and *p* = 0.001, respectively), 48 h (*p* < 0.020, *p* = 0.000, and *p* = 0.001, respectively), and 72 h (*p* = 0.008, *p* = 0.000, and *p* = 0.001, respectively) following the shock delivery.

The outcomes related to the duration spent in the illuminated area are illustrated in Figure 5e–h. Based on the results, POF rats demonstrated a notable reduction in light duration compared to the control group at 3 h, 24 h, 48 h, and 72 h following shock administration (*p* = 0.001, *p* = 0.002, *p* = 0.010, and *p* = 0.002, respectively). However, when compared to the POF group, the treatment involving probiotics, prebiotics, and synbiotics notably enhanced the duration spent in the light at 3 h (*p* = 0.008, *p* = 0.001, and *p* = 0.000, respectively), 24 h (*p* = 0.004, *p* = 0.001, and *p* = 0.000, respectively), 48 h (*p* = 0.005, *p* = 0.000, and *p* = 0.000, respectively), and 72 h (*p* = 0.008, *p* = 0.001, and *p* = 0.000, respectively) following shock delivery.

**FIGURE 6:**
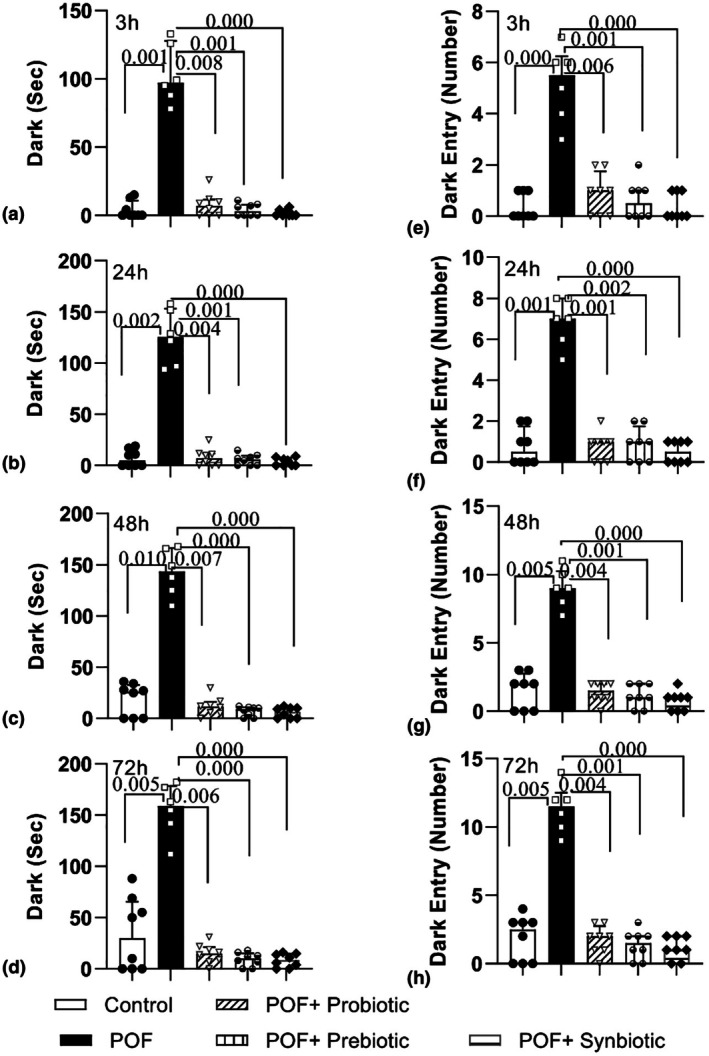
Comparison of the total time spent in the darkroom (a, b, c, d) and the number of entries to the darkroom (e, f, g, h) at the 3, 24, 48, and 72 h after receiving a shock in the passive avoidance test in the experimental groups. The data are presented as median and interquartile range (*n* = 6–8).

POF rats likewise demonstrated a notable rise in the duration spent in the dark area when compared to the control group at 3 h, 24 h, 48 h, and 72 h following shock delivery (*p* = 0.001, *p* = 0.002, *p* = 0.010, and *p* = 0.005, respectively). However, when compared to the POF group, treatment with probiotics, prebiotics, and synbiotics significantly reduced the time spent in the darkness at all assessed time intervals including 3 h (*p* = 0.008, *p* = 0.001, and *p* = 0.000, respectively), 24 h (*p* = 0.004, *p* = 0.001, and *p* = 0.000, respectively), 48 h (*p* = 0.007, *p* = 0.000, and *p* = 0.000, respectively), and 72 h (*p* = 0.006, *p* = 0.000, and *p* = 0.000, respectively) following shock delivery (Figure 6a–d).

Our findings showed that the frequency with which the animals accessed the dark zone in the POF group was notably greater than in the control group across all four time intervals: 3 h (*p* = 0.000), 24 h (*p* = 0.001), 48 h (*p* = 0.005), and 72 h (*p* = 0.005). Furthermore, the frequency with which animals in the probiotics, prebiotics, and synbiotics treatment groups accessed the dark zone indicated a notable reduction in comparison to the POF group at all assessed time intervals including 3 h (*p* = 0.006, *p* = 0.001, and *p* = 0.000, respectively), 24 h (*p* = 0.001, *p* = 0.002, and *p* = 0.000, respectively), 48 h (*p* = 0.004, *p* = 0.001, and *p* = 0.000, respectively), and 72 h (*p* = 0.004, *p* = 0.001, and *p* = 0.000, respectively) following shock delivery (Figure 6e–h).

## 4 DISCUSSION

The current research showcased the neuroprotective benefits of probiotics, prebiotics, and their synbiotic combination in addressing aversive memory deficits caused by POF, along with an enhancement in cholinergic function and attenuation of oxidative stress. Cisplatin is a well‐known anticancer drug, but it has a wide range of side effects. This anti‐tumor drug also impacts normal tissue, including the woman's reproductive system, and was therefore used to establish an animal model of POF (Li et al., 2023; Liu et al., 2023; Zhang et al., 2024). In the current research, the number of developing follicles in cisplatin‐injected groups was significantly reduced compared to the control group. Additionally, folliculogenesis was impaired, and the count of atretic follicles increased. These findings confirm that cisplatin administration successfully induced a POF model in rats.

Numerous studies have indicated that POF is associated with difficulties in learning and memory.

Based on the findings of the PA test, rats experiencing POF failed to recall the location of the shock and exhibited shorter latencies to enter the dark area at all times after the shock. They also allocated more time to the dark area and less time to the light part, with increased entries into the dark compartment. These observations align with previous research linking POF to learning and memory deficits, as well as AD (Sochocka et al., 2023; Vujović et al., 2023).

Our findings suggest that the deficits in aversive memory resulting from POF are linked to increased activity of AChE. The effects of POF on cholinergic system components, including AChE, have not been reported, but it is well known that ovarian hormones have an important role in learning and memory, and deprivation from these hormones has an important role in cognitive impairment and cholinergic dysfunction (Balderan et al., 2023; Craig et al., 2010; Hejazian et al., 2016). It was also shown that low doses of estradiol and the compounds that mimic the effect of estradiol improve cholinergic system function and learning and memory (Azizi‐Malekabadi et al., 2012; Hejazian et al., 2016; Vafaee et al., 2014; Zabihi et al., 2014).

Biochemical analyses further revealed that POF significantly exacerbated oxidative stress in the cortex, cerebellum, and hippocampus, as evidenced by elevated MDA levels and reduced thiol and SOD levels in the POF group compared to the control group. These results are consistent with earlier studies (Ağaçayak et al., 2016; Bagnall‐Moreau, 2018; Rauf et al., 2018). Oxidative damage occurs when the production of peroxides and reactive oxygen species (ROS) exceeds the capacity of the body's antioxidant defenses (Ionescu‐Tucker & Cotman, 2021; Rajabian et al., 2023). Reactive oxygen species have the potential to harm unsaturated fatty acids within brain tissue. The brain is especially susceptible to oxidative injury because of its restricted antioxidant defense systems (Ionescu‐Tucker & Cotman, 2021). Our findings suggest that the aversive memory deficits observed in this study are likely associated with oxidative stress.

This study also highlights that supplementation with probiotics, prebiotics, and their synbiotic combinations—which modulate the gut microbiome and enhance the gut–brain axis—can improve physiological and behavioral parameters, including latency, time spent in dark and light compartments, and memory performance. Previous studies have reported that these interventions protect against behavioral impairments and spatial memory deficits in AD mouse models (Kim et al., 2021). Our biochemical data indicate that probiotics, prebiotics, and their synbiotic mixtures reduced AChE activity in the hippocampus and cortex. Additionally, prebiotics and synbiotics, but not probiotics alone, prevented AChE overactivity in the cerebellum. Given the critical role of AChE in regulating acetylcholine (ACh) balance and synaptic neurotransmission, the inhibition of AChE activity by these agents suggests their potential to ameliorate cognitive impairment observed in this study (Zhang et al., 2023).

Biochemical results also revealed elevated thiol levels in the hippocampus and cortex of probiotic‐ and synbiotic‐treated groups compared to the POF group, though prebiotics were ineffective in this regard. In the cerebellum, only probiotics increased thiol content, while prebiotics and synbiotics showed no significant effects. Furthermore, probiotics, prebiotics, and synbiotics enhanced SOD activity in the hippocampus. In the cortex, probiotics and synbiotics increased SOD activity, but prebiotics did not. Interestingly, only probiotics improved SOD activity in the cerebellum, while prebiotics and synbiotics had no significant effects. These results suggest that probiotics and synbiotics may enhance antioxidant defenses in brain regions critical for learning and memory. Numerous studies have explored the free radical scavenging and antioxidant properties of probiotics, prebiotics, and their synbiotic combinations in the brain (Alam et al., 2024; Ngoc et al., 2024). Since oxidative stress is known to influence cognitive function, the improved antioxidant capacity observed in treated groups may represent an additional mechanism underlying their neuroprotective effects and memory‐enhancing properties.

In summary, our findings suggest that probiotics, prebiotics, and their synbiotic combinations exert neuroprotective effects by modulating the gut microbiome, enhancing cholinergic transmission, and reducing oxidative stress. These actions ultimately mitigate neurodegeneration associated with oxidative stress in the brain, thereby preserving memory and learning abilities. Consequently, these agents may represent a promising therapeutic approach for preventing and treating neurological disorders associated with premature ovarian insufficiency (Musa et al., 2017). As a limitation, the molecular experiment is need to be done to understand the possible mechanisms, including the signaling pathways, the neurotransmitters, and the receptors which are probably responsible for the effects of probiotics, prebiotics, and their synbiotics on learning and memory.

## 5 CONCLUSION

In summary, our findings support the idea that supplementation with probiotics, prebiotics, and their synbiotic combination positively influences the balance of the gut microbiome. This effect appears to provide protective benefits against oxidative stress‐induced brain damage, mitigate cognitive decline, and promote recovery in rats with complications arising from POF. Furthermore, this study highlights the potential therapeutic significance of probiotics, prebiotics, and synbiotics in safeguarding neurological health associated with POF and suggests promising avenues for future research and clinical applications.

## FUNDING INFORMATION

None.

## CONFLICT OF INTEREST STATEMENT

The authors declare no conflict of interest about this work.

## ETHICS STATEMENT

The research methods were granted official approval by the Ethics Committee for Animal Experimentation, following the endorsed ethical code IR. MUMS. REC. 1403. 140.

## Data Availability

All the analyzed datasets of the current study are available from the corresponding author on reasonable request.
